# Acute kidney injury burden in different clinical units: Data from nationwide survey in China

**DOI:** 10.1371/journal.pone.0171202

**Published:** 2017-02-02

**Authors:** Xiaojing Tang, Dongping Chen, Shengqiang Yu, Li Yang, Changlin Mei, on behalf of ISN AKF 0 by 25 China Consortium

**Affiliations:** 1 Division of Nephrology, Shanghai Changzheng Hospital, Second Military Medical University, Shanghai, China; 2 Renal Division, Peking University First Hospital, Beijing, China; Thomas Jefferson University, UNITED STATES

## Abstract

**Background:**

The inpatient morbidity and mortality of acute kidney injury (AKI) vary considerably in different clinical units, yet studies to compare the difference remain limited.

**Methods:**

We compared the clinical characteristics of AKI in Intensive Care Unit (ICU), medical and surgical departments by using the data derived from the 2013 nationwide cross-sectional survey of AKI in China to capture variations among different clinical departments in recognition, management, and outcomes of AKI. Suspected AKI patients were identified based on changes in serum creatinine during hospitalization, and confirmed by reviewing medical records.

**Results:**

The detection rate of AKI was the highest in ICU (22.46%), followed by the rates in medical (1.96%) and surgical departments (0.96%). However, the absolute number of cases was the largest in medical departments, which contributed to 50% of the cases. In medical departments, 78% of AKI cases were extensively distributed in cardiac, nephrology, oncology, gastroenterology, pneumology and neurology departments. In contrast, 87% of AKI cases in surgical departments were mainly from urology, general surgery and cardiothoracic departments. The in-time recognition rates were extremely low in all departments except nephrology. Only 10.5~15.0% AKI patients from non-nephrology departments received renal referral. Among all the death cases, 50% and 39% came from ICU and medical departments while only 11% from surgical departments. Older age, higher AKI stage and renal replacement therapy indication were identified as risk factors for high mortality in all departments. Delayed recognition and no renal referral were significantly associated with increased mortality in medical and ICU patients.

**Conclusions:**

These findings suggest that ICU and medical departments are major affected departments in China with a large number of AKI cases and subsequent high mortality. The reality is more alarming considering the low awareness of AKI and the paucity of effective interventions in the high-risk patients in these departments.

## Introduction

Acute kidney injury (AKI) is a world-wide common clinical problem. In 2013, the International Society of Nephrology (ISN) launched a global initiative of “0 by 25” project to achieve the goal of zero death of patients with untreated AKI by 2025, with the purpose to reduce the enormous growing burden of AKI and its consequences. So far, various epidemiology studies from the developed countries showed the incidence of AKI was about 3–18%[1, 2]. The latest national survey from China reported the detection rate of AKI was 2.03% and all-cause mortality rate was 12.4%[3]. The common issue found in the study was the extremely low recognition and renal referral rate of AKI, especially in the non-nephrology departments[3]. Despite multiple epidemiology studies in a variety of populations, the major burden and key affected clinical units of AKI among the hospitalized population remain unclear. In this study, we analyzed the clinical characteristics of AKI in different clinical units using the data from nationwide AKI survey in China for the purpose of capturing variations among different clinical departments in distribution, etiology, recognition, and outcomes of AKI.

## Materials and methods

### Study participants and data collections

The data we used was derived from the 2013 nationwide cross-sectional survey of AKI in China which included 22 provinces, municipalities and autonomous regions, where covering 82% of the country’s population and the four geographical regions of China[3]. On the basis of the available research manpower, patients who were hospitalized in two individual months, January 2013 and July 2013, were included. The study, including the use of anonymous data, was approved by the Ethics committee of Peking University First Hospital.

The diagnostic criteria included two, the 2012 KDIGO AKI definition[4] (criteria 1) and the expanded criteria. For those who had no repeated serum creatinine (SCr) measurement within 7 days or with recovering AKI, the expanded criteria was: an increase or decreases in SCr by 50% during hospital stay [3] (criteria 2). Patients who had CKD stage 5, nephrectomy, kidney transplantation, or peak SCr<0.6mg/dl, were excluded. Those who met the identification criteria but the SCr change could not be attributed to AKI were also excluded. Suspected AKI patients were identified based on changes in serum creatinine during hospitalization, and confirmed by reviewing medical records. Detailed hospital selection, survey protocol and screening flow diagram were reported in the previous published paper by LY[3].

The detected AKI cases were investigated by the nephrologists to record the clinical departments, comorbidities, diseases or conditions that could cause renal hypoperfusion or urinary obstruction, nephrotoxic medications and environmental nephrotoxins, invasive procedures and surgeries, critical illness, AKI classification, renal replacement therapy, renal referral and all-cause in-hospital death. Renal recovery at discharge was determined as: full recovery, serum creatinine (Scr) fell below threshold or to the baseline; partial recovery, Scr decreased by ≥25% from peak level but remained above the threshold or baseline; failure to recover, dialysis dependent or Scr decreased by <25% from peak level.

“Recognition of AKI by the physicians-in-charge” was defined if there were any medical records of increased creatinine levels, concerns about the deterioration of the kidney function, otherwise non-recognition was defined. Recognition rate referred to the percent of AKI patients who were recognized by their physicians-in-charge. Timely recognition was defined if AKI was recognized by the physicians-in-charge within three days after AKI could be diagnosed and before progressed to higher stages, otherwise delayed recognition was defined.

### Statistical analysis

The detection rates of AKI were calculated by number of detected AKI cases/number of admission. Continuous data were presented as means with SDs or median with interquartile ranges as appropriate. Categorical variables were presented as proportions. The characteristics of patients and the statues of recognition and treatment of AKI were described and stratified by clinical departments, including medical, surgical and ICU departments. Comparisons among groups were conducted using one-way ANOVA or Kruskal-Wallis test for continuous variables and Chi-square test for categorical variables. Relevant covariates that might associate with all-cause in-hospital mortality in various departments were analyzed with the multivariable logistic regression and odds ratios (ORs) with 95% confidence intervals (CIs) and P values of Wald Chi-square test were reported. Covariates included in the analysis were age (change by 10 years), gender (male vs female), Choric kidney disease (yes vs no), cardiovascular disease (yes vs no), diabetes (yes vs no), hypertension (yes vs no), renal referral (yes vs no), AKI stages at detection, Renal replacement therapy indication (yes vs no), and recognition of AKI (non-recognition, delayed recognition, timely recognition). The cases with missing information of the covariates were excluded in the logistic regression.

We used Epidata software (version 3.1, Epidata Association, Odense, Denmark) for data entry and management. All P values are two-sided, and a P value of less than 0.05 was considered significant. Analyses were done with SAS software (version 9.1, SAS Institute, Cary, NC)

## Results

### Distribution of AKI cases in different clinical settings

**Fig 1:**
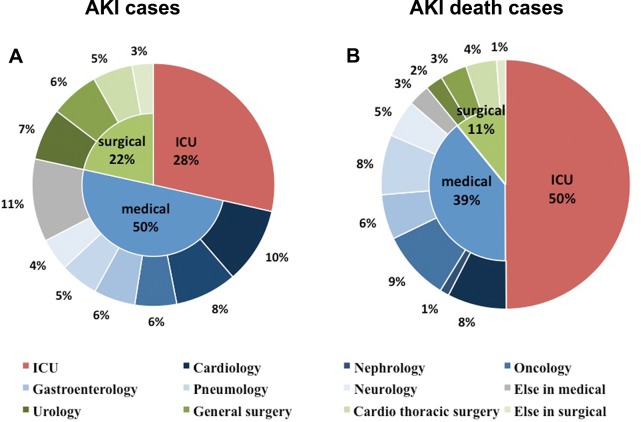
The distribution of AKI cases and AKI death cases in different clinical departments. Panel A showed the distribution of AKI cases. Among all the AKI patients, 50% were from medical departments, 29% from ICU and 21% from surgical departments. Panel B displayed the distribution of AKI death cases. ICU contributed to half of the death cases.

During the two months of January 2013 and July 2013, there were 374,286 admissions in which 7,604 were detected as AKI based on KDIGO and the expanded criteria. As shown in **Table 1**, the detection rate of AKI was the highest in intensive care unit (ICU) (22.46%), followed by the rates in medical (1.96%) and surgical departments (0.96%). However, the absolute number of cases was the largest in medical departments, which contributed to 50% of the cases (**Fig 1**). In medical departments, 78% of AKI cases were extensively distributed in cardiac, nephrology, oncology, gastroenterology, pneumology and neurology departments. Unlike the broad distribution of AKI among medical departments, AKI cases were mainly from urology, general surgery and cardiothoracic departments, which accounted for 87% of all the AKI patients in surgical departments. There were 64.9% of AKI episodes in ICU detected by KDIGO criteria while only 41.7~46.1% identified in medical and surgical departments.

**Table 1:** The detection rates of AKI and characteristics of patients in medical, surgical and ICU departments^*^.

	Patients from medical departments (n = 3796)	Patients from surgical departments (n = 1639)	Patients from ICU (n = 2169)	P value
Age	62.4±18.3	59.7±15.3	61.6±17.2	<0.001
Age group				<0.001
18~39	483(12.7%)	154(9.4%)	239(11.0%)	
40~59	1035(27.3%)	630(38.4%)	676(31.2%)	
60~79	1508(39.7%)	695(42.4%)	917(42.3%)	
≥80	770(20.3%)	160(9.8%)	337(15.5%)	
Gender	2366(62.3%)	1152(70.3%)	1437(66.3%)	<0.001
Detection rate (n (%))				
Hospital admission	193183	171446	9657	
KDIGO	1554(0.80%)	741(0.43%)	1392(14.41%)	<0.001
KDIGO+ΔSCr≥50%	3796(1.96%)	1639(0.96%)	2169(22.46%)	<0.001
AKI stage				<0.001
1	1832(48.2%)	828(50.5%)	823(37.9%)	
2	940(24.8%)	377(23.0%)	633(29.2%)	
3	1024(27.0%)	434(26.5%)	713(32.9%)	
Recognition rate				<0.001
Unrecognized	2677(70.9%)	1359(83.5%)	1572(73.0%)	
Delayed	176(4.7%)	59(3.6%)	108(5.0%)	
In-time	922(24.4%)	209(12.9%)	473(22.0%)	
AKI causes				
CA-AKI	2267(59.7%)	886(54.1%)	983(45.3%)	<0.001
Pre-renal	1863(49.1%)	794(48.4%)	1279(59.0%)	<0.001
Renal	1233(32.5%)	262(16.0%)	605(27.9%)	<0.001
Post-renal	198(5.2%)	397(24.2%)	75(3.5%)	<0.001
Unclassified	502(13.2%)	186(11.3%)	210(9.7%)	<0.001
Risk factors				
Renal hypoperfusion	2955(77.8%)	1094(66.7%)	1865(86.0%)	<0.001
Nephrotoxic drugs	2718(71.6%)	1099(67.1%)	1627(75.0%)	<0.001
Environmental toxins	125(3.3%)	14(0.9%)	52(2.4%)	<0.001
Sepsis	166(4.4%)	73(4.5%)	244(11.3%)	<0.001
Other critical illness	1191(31.4%)	618(37.7%)	1415(65.2%)	<0.001
Comorbidity				
CKD	1163(30.6%)	315(19.2%)	369(17.0%)	<0.001
HBP	1683(44.3%)	538(32.8%)	969(44.7%)	<0.001
DM	800(21.1%)	180(11.0%)	424(19.5%)	<0.001
CVD	1433(37.8%)	315(19.2%)	918(42.3%)	<0.001
Malignancy	711(18.7%)	424(25.9%)	283(13.0%)	<0.001
RRT indication	414(10.9%)	104(6.3%)	378(17.4%)	<0.001
Renal referral rate	1091(28.7%)	192(11.7%)	342(15.8%)	<0.001
Renal recovery at discharge				0.87
Complete recovery	1023(32.8%)	447(32.1%)	577(32.3%)	
Partial recovery	1032(33.0%)	466(33.4%)	573(32.1%)	
Non-recovery	1068(34.2%)	481(34.5%)	635(35.6%)	
Mortality	363(9.7%)	101(6.3%)	463(21.8%)	<0.001

Abbreviations: CKD, chronic kidney disease; HBP, hypertension; DM diabetic mellitus; CVD cardiac vascular disease; RRT, renal replacement therapy.

* 49 cases missing the information for recognition rate, 1 for CVD, 1302 for renal recovery at discharge and 129 for mortality.

### Clinical characteristics of AKI in different clinical settings

The baseline characteristics and risk factors for AKI in ICU, medical and surgical departments were displayed in Table 1. Patients in medical and ICU departments were older than those in surgical departments. There were 20.3% of patients over 80 years old in medical departments, compared with 15.5% in ICU and 9.8% in surgical departments. Male patients were more commonly seen in surgical departments than those in medical and ICU departments. Most of the AKI patients in the medical and surgical departments were in stage 1 and stage 2, while a greater proportion of AKI patients (32.9%) from ICU were in stage 3. This corresponded to the actual clinical situation. Patients in ICU and medical departments were significantly more likely to have co-morbidities, including hypertension, diabetes and cardiac vascular diseases. Risk factors including renal hypoperfusion, use of nephrotoxic drugs, sepsis and other critical disease were most frequently seen in patients from ICU, followed by medical departments. More than half of patients in medical and surgical departments were caused by community acquired AKI (CA-AKI) whereas 45.3% in ICU suffered CA-AKI. Accordingly, pre-renal causes contributed to 59% of the AKI cases in ICU, which were more common than those in medical and surgical departments. The in-time recognition rates were extremely low in all departments except nephrology. Identically, only 10.5~15.0% of AKI patients from non-nephrology departments received renal referral.

In the sub-analysis of the medical and surgical departments, we found interesting similarity of the clinical features in the special departments related to the same organ, such as kidney and heart (**S1 Table**). There were 52.5% and 40.3% of the cases reaching AKI stage 3 in nephrology and urology departments (kidney related departments) while only 19% had AKI stage 3 in cardiology and cardiothoracic surgery departments (cardiac related departments). Yet, the mortality rate was much higher in the patients from cardiac related departments than those from kidney related departments. The proportion of CA-AKI was much higher in kidney related departments (82% in nephrology and 72.2% in urology) than the cardiac related departments (49.9% in cardiology and 24.1% in cardiothoracic surgery). There were much more pre-renal AKI cases in cardiac related departments than those in kidney related departments. About 90% of patients with AKI from cardiac related departments were found with the risk factor of renal hypoperfusion. Although nephrotoxins were prevalent in all departments, it was least commonly used in kidney related departments.

### Outcomes in different clinical settings

**Fig 2:**
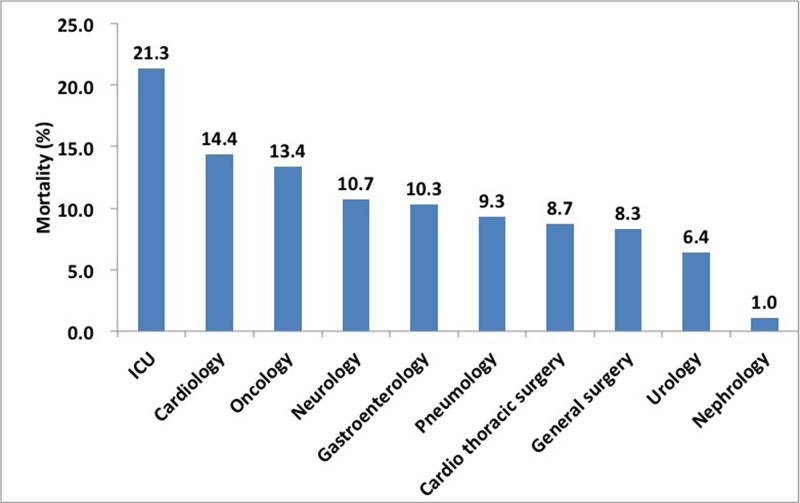
Mortality of AKI in different clinical departments.

The all-cause in-hospital mortality rate in ICU was 21.8%, which was highest among all the clinical units (**Fig 2**). Among all the death cases of AKI, 50% and 39% of the cases came from ICU and medical departments while only 11% from surgical departments **(Fig 1**). About 77% of death cases in medical departments came from cardiac, oncology, pneumology and gastroenterology departments. Conversely, only 1.8% and 4.0% of AKI patients died in nephrology and urology department respectively. There was no significant difference of renal survival among the various clinical units. In multivariate analysis, older age, higher AKI stage and renal replacement therapy (RRT) indication were identified as risk factors for high mortality in all departments. Delayed recognition and no renal referral were significantly associated with increased in-hospital mortality for medical and ICU patients (**Table 2**).

**Table 2:** Multivariate logistic regression analysis for factors associated with all-cause in-hospital mortality in AKI in different clinical departments*.

	Patients from medical departments (n = 3724)		Patients from surgical departments (n = 1602)		Patients from ICU(n = 2105)	
	OR (95%CI)	P	OR (95%CI)	P	OR (95%CI)	P
Age (per 10 years)	1.4(1.3,1.6)	<0.001	1.2(1.0,1.4)	0.03	1.3(1.2,1.4)	<0.001
Sex (male vs female)	1.9(1.5,2.5)	<0.001	1.6(1.0,2.6)	0.07	1.0(0.8,1.3)	0.98
History of CVD (yes vs no)	0.9(0.7,1.2)	0.64	2.2(1.3,3.5)	0.002	1.0(0.8,1.2)	0.78
DM (yes vs no)	1.3(1.0,1.7)	0.08	0.9(0.5,1.8)	0.83	0.9(0.7,1.2)	0.42
CKD (yes vs no)	0.9(0.7,1.2)	0.49	0.5(0.3,0.9)	0.03	1.2(0.9,1.6)	0.16
HBP (yes vs no)	0.8(0.6,1.0)	0.07	1.0(0.6,1.7)	0.88	1.2(1.0,1.5)	0.11
AKI stage at peak						
AKI 1	Reference		Reference		Reference	
AKI 2	2.1(1.6,2.8)	<0.001	3.0(1.7,5.4)	<0.001	2.2(1.7,3.0)	<0.001
AKI 3	3.0(2.2,4.0)	<0.001	4.5(2.5,8.0)	<0.001	3.3(2.5,4.5)	<0.001
RRT indication (yes vs no)	2.6(1.9,3.7)	<0.001	2.3(1.2,4.5)	0.02	1.7(1.3,2.3)	<0.001
Renal referral (yes vs no)	0.5(0.4,0.7)	<0.001	1.4(0.8,2.6)	0.24	0.6(0.5,0.9)	0.004
Timely recognition vs. Delayed recognition	0.4(0.3,0.7)	<0.001	0.6(0.2,1.7)	0.36	0.6(0.3,0.9)	0.01

Abbreviations: CI confidence interval.

*The number of cases included in the analysis were listed in the table after excluding the cases with missing information.

## Discussion

AKI is a common clinical problem, affecting 2–22% of all patients admitted to hospital[1, 5, 6]. However, the inpatient morbidity and mortality of AKI varies considerably in different clinical units[1]. Besides, a lot of patients are usually under the care of specialists from departments other than nephrology, who may not always be familiar with the optimum care of patients with AKI. In 2009, the National Confidential Enquiry into Patient Outcome and Death (NCEPOD)[7] reported only 50% of patients who died from AKI had received 'good' care. Furthermore, for 20% of these patients, the cause was both predictable and preventable. These results may suggest that current strategies to reduce and prevent AKI are ineffective. AKI associated with different conditions may have different clinical features and require specific management strategies. Despite ample observational studies about AKI in specific clinical settings such as ICU[8–10] and cardiac surgery[11, 12], comparison of the features of AKI in different clinical setting remains relatively limited. Our observations could help to highlight the characteristics of major affected patient groups, and to provide information for the reference of clinical decision-making and optimization of intervention strategy.

ICU and operative settings were always regarded as the major contribution to AKI patients in the previous AKI studies[8, 13, 14]. It is no doubt that patients in ICU always presented with higher severity of AKI, more complicated critical illness, increased in-hospital mortality rate and higher medical cost[1]. The recent multinational AKI-EPI study reported AKI occurred in 57% of the ICU patients[8]. Our study also showed the detection rate of AKI was highest in ICU among all the clinical units with the highest mortality. However, besides ICU we found half AKI cases were detected from medical departments which had the largest number of patients among the whole hospitalized population. AKI cases were dispersively distributed in cardiac, nephrology, oncology, pneumology and gastroenterology departments. Among the 3796 AKI patients from medical departments, only 627 cases came from nephrology department. In contrast, there were fewest AKI cases in surgical departments with the lowest mortality. Actually, medical departments and ICU contributed to 70% of the AKI cases instead of nephrology and surgical wards. AKI therefore represents an important burden in the medical departments for health care as well. Furthermore, the scattered distribution of AKI cases in medical departments suggested a much more challenging and tougher situation to manage.

Another notable factor in the burden of AKI in hospitalized patients is poor general knowledge about the important role of the kidney and the absence of recognition in the non-kidney departments. Hospital acquired AKI (HA-AKI) was found to be the most common cause of AKI in almost all non-kidney departments especially in ICU and cardiac related departments. The latter finding suggests that initial hospital care in these departments often fails to prevent AKI. HA-AKI was proved to be associated with a much higher mortality compared to CA-AKI[15]. The development of HA-AKI may reflect delayed referral to and involvement of the nephrology team. A pilot study showed early nephrologists involvement would improve the outcomes of HA-AKI[16], while delayed nephrology referral is linked to higher mortality and dialysis dependence in AKI patients[5, 17]. The in-time recognition rate for AKI was lower than 25% in all departments except nephrology. The large gap in AKI recognition between nephrologists and other departments suggests that there is a dearth of understanding of the disorder among physicians. A survey made among the physicians from developing countries showed the major barriers to raise awareness for AKI were inadequate training of health workers, limited access to health care facilities and lack of support for AKI programs from stakeholders[18]. To tackle this issue we recognize that a multifaceted approach will be necessary, encompassing education and training in the adequate assessment of risk factors, early recognition and timely prevention of AKI, and need for early involvement of nephrologists where possible.

AKI associated with different conditions may require different management strategies. Although renal hypoperfusion, nephrotoxic drugs, infections were all important aetiological factors for AKI, the proportions of these precipitating factors across the different clinical settings probably reflect different exposures. The higher incidence of renal hypoperfusion and nephrotoxic agents use in patients in heart related departments might reflect exposure to several diagnostic (eg, contrast imaging) and therapeutic interventions in patients with heart diseases. By contrast, in ICU, renal hypoperfusion, sepsis, critical illnesses and more co-morbidities were the most important drivers of AKI.

Interestingly, our study found the departments related to the same organ such as heart and kidney had similar clinical features and outcome of AKI. Although there were over 40% patients reaching AKI stage 3 in nephrology and urology department, AKI in these patients represented as relatively low mortality compared to the cases in other departments. A single-center observational study from UK[19]also reported overall 30-day mortality in the total hospitalized urology population was lower than seen in studies of other AKI populations. In contrast, the majority of AKI cases in cardiac related departments were in stage 1, yet the mortality was much higher. The relatively good outcome in kidney-related department might be partially explained by a greater proportion of selective patients in these departments, which is in contrast to AKI patients in other clinical departments such as cardiac departments where AKI occurs as part of concomitant severe heart disease or after major surgery and as ICU where over 90% are admitted as an emergency or with critical illnesses. Nevertheless, the specialty and stronger awareness of AKI in nephrologists and urologists is certainly helpful to improve the outcome of AKI, which can be illustrated by the fact of less renal injury factors including nephrotoxins and renal hypoperfusion and higher recognition rate of AKI. The use of nephrotoxins was seen in over 75% of our patients in the heart related departments. In the developed countries, 30~45% of patients experienced a potential adverse drug event[9, 20]. The preexisting pre-renal state in the patients from cardiac departments may be exacerbated by the use of medications that impair the autoregulation of renal blood flow[12].

The national survey in China showed 51.5% of the AKI patients were missed by the KDIGO criteria and detected by the expanded criteria instead [3]. KDIGO criteria seemed to be more sensitive to the patients in ICU and recognized 70% of the AKI cases. In contrast, over 50% of patients were missed in medical and surgical departments by KDIGO criteria. This result suggested longer observation window should be taken for the patients in these departments.

There are several limitations of this study. First, AKI was defined according to criteria based on serum creatinine independently of urinary output, which would have further underestimated the detection rate of AKI. Second, this study represents a snapshot in time, this may have led to sampling bias. Third, disease entities in some of the medical departments such as geriatric department and oncology department were quite variable in different hospitals in China. There could be misclassification in these departments. However, the similarity of the disease entities in the major clinical departments ensures that our results are still widely generalizable.

Strengths of this study include its large nationally study population from the regions where covered 82% of China’s population, identification of AKI through laboratory values, and careful evaluation of incidence, risk factors, and outcomes across different departments. The results provide novel information for the health authorities about the major affected clinical settings of AKI and key problems during the management, which may be helpful for the AKI prevention in developing countries like China. In addition, our results suggest that AKI risk and prognosis may be similar in the setting of disease localized to the same organ. This can be used to both predict the risk of events and develop specific preventive strategies for the patients involved with the same organ.

In summary, ICU and medical departments instead of surgical departments are major affected departments in China with a large number of AKI cases and subsequent high mortality. The reality of AKI is even more alarming considering the low awareness of AKI in the majority of non-kidney clinical units and the paucity of effective interventions to prevent AKI in the high-risk patients in these departments. Deficiencies in the care of these patients included failures in AKI prevention, recognition, therapy and timely access to nephrologists and extremely common use of nephrotoxins. We expect that the delineation of the characteristics in different clinical settings could help to guide efforts for defining AKI prevention and treatment strategies.

## Supporting information

S1 Table Characteristics of AKI in kidney and cardiac related departments. (DOCX)
