# Supplementary material for: Acute kidney injury burden in different clinical units: Data from nationwide survey in China
Source: PLoS One. 2017 Feb 2;12(2):e0171202. doi: 10.1371/journal.pone.0171202 (PMC5289568; doi:10.1371/journal.pone.0171202)
Supplement: S1 Table — (DOCX) [file pone.0171202.s001.docx]

S1 Table. Characteristics of AKI in kidney and cardiac related departments*

|  | Nephrology department (n=627) | Cardiology department (n=771) | Urology department (n=526) | Cardiothoracic surgery department (n=403) | P |
| --- | --- | --- | --- | --- | --- |
| Detection rate (n (%)) | | | | | |
| Hospital admission | 43875 | 87194 | 49887 | 31949 |  |
| KDIGO（%） | 233(0.53%) | 383(0.44%) | 201(0.40%) | 239(0.75%) | <0.001 |
| KDIGO+ΔSCr≥50% | 627(1.43%) | 771(0.88%) | 526(1.05%) | 403(1.3%) | <0.001 |
| AKI stage | | | | | <0.001 |
| 1 | 183(29.2%) | 430(55.8%) | 213(40.5%) | 232(57.6%) |  |
| 2 | 115(18.3%) | 192(24.9%) | 101(19.2%) | 94(23.3%) |  |
| 3 | 329(52.5%) | 149(19.3%) | 212(40.3%) | 77(19.1%) |  |
| Recognition rate | | | | | <0.001 |
| Unrecognized | 190(30.4%) | 606(79.2%) | 381(73.3%) | 348(87.0%) |  |
| Delayed | 27(4.3%) | 41(5.4%) | 17(3.3%) | 13(3.25%) |  |
| In-time | 408(65.3%) | 118(15.4%) | 122(23.4%) | 39(9.75%) |  |
| AKI causes | | | | |  |
| CA-AKI | 514(82.0%) | 385(49.9%) | 380(72.2%) | 97(24.1%) | <0.001 |
| Pre-renal | 174(27.8%) | 494(64.1%) | 75(14.3%) | 265(65.8%) | <0.001 |
| Renal | 360(57.4%) | 176(22.8%) | 56(10.6%) | 78(19.4%) | <0.001 |
| Post-renal | 49(7.8%) | 21(2.7%) | 354(67.3%) | 11(2.7%) | <0.001 |
| Unclassified | 44(7.0%) | 80(10.4%) | 41(7.8%) | 49(12.1%) | 0.02 |
| Risk factors | | | | |  |
| Renal hypoperfusion | 443(70.7%) | 723(93.8%) | 237(45.1%) | 352(87.3%) | <0.001 |
| Nephrotoxic drugs | 399(63.6%) | 599(77.7%) | 318(60.5%) | 331(82.1%) | <0.001 |
| Environmental toxins | 30(4.8%) | 17(2.2%) | 2(0.4%) | 2(0.5%) | <0.001 |
| Sepsis | 20(3.2%) | 10(1.3%) | 23(4.4%) | 17(4.2%) | 0.004 |
| Other critical illness | 82(13.1%) | 210(27.2%) | 137(26.0%) | 215(53.3%) | <0.001 |
| Comorbidity | | | | |  |
| CKD | 341(54.4%) | 255(33.1%) | 172(32.7%) | 52(12.9%) | <0.001 |
| HBP | 258(41.1%) | 479(62.1%) | 149(28.3%) | 129(32.0%) | <0.001 |
| DM | 83(13.2%) | 189(24.5%) | 49(9.3%) | 40(9.9%) | <0.001 |
| CVD | 96(15.3%) | 621(80.5%) | 46(8.7%) | 215(53.3%) | <0.001 |
| Malignancy | 30(4.8%) | 37(4.8%) | 107(20.3%) | 86(21.3%) | <0.001 |
| RRT indication | 171(27.3%) | 72(9.3%) | 53(10.1%) | 29(7.2%) | <0.001 |
| Renal referral rate | 627(100.0%) | 116(15.0%) | 55(10.5%) | 51(12.7%) | <0.001 |
| Mortality | 11(1.8%) | 71(9.3%) | 21(4.1%) | 37(9.3%) | <0.001 |
| Renal recovery at discharge |  | | | | 0.94 |
| Complete recovery | 172(32.8%) | 199(31.2%) | 151(34.4%) | 100(30.8%) |  |
| Partial recovery | 170(32.4%) | 211(33.1%) | 140(31.9%) | 111(34.2%) |  |
| Non-recovery | 182(34.7%) | 227(35.6%) | 148(33.7%) | 114(35.1%) |  |

* 17 cases missing the information for recognition rate, 402 for renal recovery at discharge and 41 for mortality.
